# Mutational and transcriptional analyses of an avian pathogenic *Escherichia coli *ColV plasmid

**DOI:** 10.1186/1471-2180-8-24

**Published:** 2008-01-29

**Authors:** Jerod A Skyberg, Timothy J Johnson, Lisa K Nolan

**Affiliations:** 1 Department of Veterinary Microbiology and Preventive Medicine, College of Veterinary Medicine, Iowa State University, Ames, IA 50011, USA; 2 Department of Veterinary Molecular Biology, Montana State University, Bozeman, MT 59718, USA; 3 Department of Veterinary and Biomedical Sciences, University of Minnesota, St. Paul, MN 55108, USA

## Abstract

**Background:**

Previously we described a 184-kb ColV plasmid, pAPEC-O2-ColV, that contributed to the ability of an *E. coli *to kill avian embryos, grow in human urine, and colonize the murine kidney. Here, the roles of several genes encoded by this plasmid in virulence were assessed using mutational and transcriptional analyses.

**Methods:**

Genes chosen for deletion were *iss*, *tsh*, *iutA*, *iroN*, *sitA*, and *cvaB*. In addition, a 35-kb region of the plasmid, containing *iss*, *tsh*, and the ColV and *iro *operons, along with a 15-kb region containing both the aerobactin and *sit *operons, were deleted. Mutants were compared to the wild-type (APEC O2) for lethality to chick embryos and growth in human urine. Expression of the targeted genes was also assessed under these same conditions using RT-PCR

**Results:**

No significant differences between the mutants and the wild-type in these phenotypic traits were detected. However, genes encoding known or predicted iron transport systems were up-regulated during growth in human urine, as compared to growth in LB broth, while *iss*, *hlyF*, and *iroN *were strongly up-regulated in chick embryos.

**Conclusion:**

While no difference was observed between the mutant strains and their wild-type parent in the phenotypic traits assayed, we reasoned that some compensatory virulence mechanism, insensitivity of the virulence assays, or other factor could have obscured changes in the virulence of the mutants. Indeed we found several of these genes to be up-regulated in human urine and/or in the chick embryo, suggesting that certain genes linked to ColV plasmids are involved in the establishment of avian extraintestinal infection.

## Background

A 184-kb ColV plasmid, known as pAPEC-O2-ColV, was sequenced and analyzed [1]. In addition to regions devoted to plasmid transfer, maintenance, and replication, pAPEC-O2-ColV was found to contain a 94-kb putative pathogenicity island (PAI), containing *hlyF*, *ompT*, *iss*, *tsh*, the ColV operon, and several genes encoding known or predicted iron transport systems. The iron-related systems included those encoding aerobactin and salmochelin, and the *sit *ABC transport system. Additionally, pAPEC-O2-ColV contained a putative iron transport system novel to APEC called *eit *and another putative ABC transport system known as *ets*. This plasmid was transmissible by conjugation from the donor, avian pathogenic *Escherichia coli *(APEC) O2, to recipient strains, and it was found to co-transfer with a large R plasmid known as pAPEC-O2-R [2]. When the role of APEC O2's plasmids in virulence was investigated, it was found that acquisition of these plasmids resulted in an enhancement in the recipient's ability to kill avian embryos, grow in human urine, and colonize the murine kidney [3]. It was thought that the increase in virulence was likely due to acquisition of pAPEC-O2-ColV [3].

Also, a study of the distribution of genes of pAPEC-O2-ColV's putative PAI in APEC and avian fecal commensal *E. coli *(AFEC) isolates revealed that a portion of this PAI was highly conserved among APEC and that these conserved genes occurred much more often in APEC than in commensal strains [4,5]. This conserved portion, which occurred in most of the APEC examined, included *sit*, an iron/manganese transport system [6,7]; salmochelin and aerobactin, both siderophore iron acquisition systems [8,9]; *ets*, a putative ABC transport system [1]; *hlyF*, an avian hemolysin [10]; *iss*, the increased serum survival gene [11]; *ompT*, an outer membrane protease [12]; the RepFIB replicon; and the 5' end of the ColV operon [1,4]. The variable portion of this PAI contained the 5' end of the ColV operon; *tsh*, the temperature sensitive hemagglutinin gene [13,14]; and the *eit *operon [1]. The split between conserved and variable portions occurred within the *cvaB *gene of the ColV operon, with the 5' end of *cvaB *and many of its upstream genes occurring significantly more often among APEC than the 3' end of *cvaB *and many of its downstream genes [1,4]. Genes of these plasmid-linked PAIs occur widely among APEC isolated from different parts of the world [5,15-20] various avian host species [5,15,20] and different syndromes [5,21]. These observations suggest that these plasmid-linked PAIs, especially their conserved portions, might be a defining characteristic of the APEC pathotype [5] that could be exploited in colibacillosis control. Indeed, protocols for rapid characterization of APEC based on detection of certain virulence genes, including some from this cluster, show promise [22,23].

While the data from these epidemiological studies are useful at identifying genes of interest and have been widely used to characterize avian *E. coli *[5,15,22,23], they are no substitute for an in-depth study of the contributions to virulence of individual genes. Such studies typically require comparisons of wild-type and mutant strains, differing in a single trait of interest, for their abilities to cause disease in animal models [24]. Here, we sought to determine the contributions of certain genes of pAPEC-O2-ColV's PAI to *E. coli*'s ability to kill embryos and grow in human urine using mutational analysis. When differences in virulence between the mutants and the wild-type were not detected, follow-up studies to determine if these same genes are differentially expressed in APEC O2 during infection were undertaken.

## Results and Discussion

Large conjugative ColV plasmids and the genes they carry are found in a much higher proportion of *E. coli *incriminated in cases of avian colibacillosis than in *E. coli *isolated from the feces of apparently healthy birds [5,25]. In addition, it has been shown that ColV plasmids may mediate avian *E. coli *virulence and are often implicated in cases of human extraintestinal disease [3,26-28]. In a previous study we found that transfer of a large ColV plasmid, pAPEC-O2-ColV (along with a co-transferring R plasmid) conferred upon a recipient strain enhanced abilities to kill chick embryos, grow in human urine, and cause urinary tract infection (UTI) in mice [3]. In the present study, we sought to determine what regions of this ColV plasmid contributed to these traits.

To do this, we mutated several genes localized to pAPEC-O2-ColV. The genes chosen for mutagenesis, *iss*, *tsh*, *cvaB*, *iutA*, *iroN*, and *sitA*, have been found to be epidemiologically associated with APEC [1,5]. In addition *tsh *and *iroN*, have been shown to contribute to the virulence of APEC [28,29], while virulence of *E. coli *K-12 was shown to be enhanced after the acquisition of an *iss *encoding plasmid [11]. The expected mutations were verified by PCR protocols [30] targeting the deleted gene and the new kanamycin resistance (*kan*^R^) cassette junction fragment. To ensure that the mutants were truly isogenic, we examined the genotype of the mutant strains for over 40 other virulence associated genes and allelic variants using published protocols [5,12,23]. In no instance did we find a loss of a gene other than the one which was targeted. This observation suggests that the method of mutagenesis used in this study may have a higher fidelity than does suicide vector driven allelic exchange which has been found to produce secondary mutations at a high rate in extraintestinal pathogenic *E. coli *[31].

**Figure 1:**
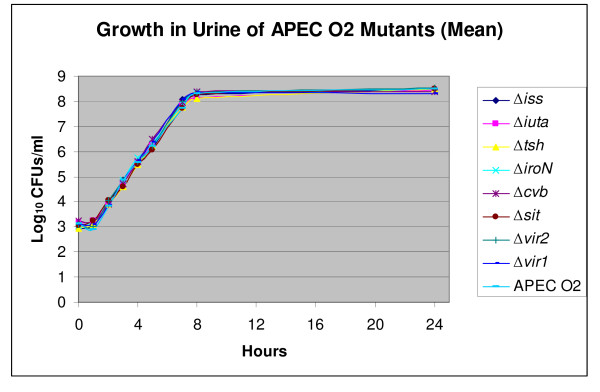
Growth in urine of APEC O2 (the source of the pAPEC-O2-ColV) and its mutant derivatives. Growth curves were determined by measuring viable counts (CFU ml^1^) and reflect an average of two trials for each strain.

**Figure 2:**
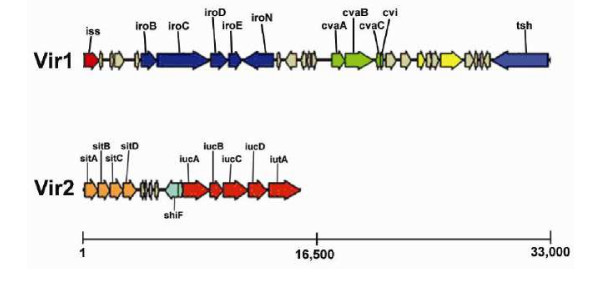
Genetic map of Vir1 and Vir2 regions of pAPEC-O2-ColV, drawn to scale with ruler in base pairs.

After confirming that our mutants were isogenic, their relative virulence, as compared to the wild type, was compared in two models, including those based on lethality to chick embryos and ability to grow in human urine. None of the mutants showed any attenuation in virulence as compared to the wild-type parent (Table 1), and none deviated from the wild-type pattern of growth in urine (Figure 1). Reasoning that the virulence of APEC is multifactorial [5,32] and that the effects of single gene knock-outs might be obscured by some compensatory mechanism, we subsequently deleted two large regions of pAPEC-O2-ColV to create deletions of Vir1 and Vir2. Region Vir1 is about 33 kb in length and contains the *iss *and *tsh *genes along with ColV and *iro *operons, while region Vir2 is around 15 kb in length and contains the *sit *and aerobactin operons (Figure 2). Deletion of these two regions also caused no attenuation of APEC O2's virulence for chick embryos (Table 1).

**Table 1:** Embryo Lethality of APEC O2 along with its Mutant Derivatives

Strain	Embryos Inoculated	Deaths	Embryo Lethality %	Z value^A^	p-value
NC	41	1	2.4	6.60**	< 0.01^A^
APEC O2	41	30	73.2	-	-
APEC 02Δ*iss*	41	29	70.7	0.25	0.81
APEC 02Δ*iutA*	41	28	68.3	0.49	0.63
APEC 02Δ*tsh*	41	32	78.0	0.51	0.61
APEC 02Δ*iroN*	41	30	73.2	0.00	1.00
APEC 02Δ*cvaB*	41	29	70.7	0.25	0.81
APEC 02Δ*sitA*	41	29	70.7	0.25	0.81
APEC 02Δ*vir1*	41	28	68.3	0.49	0.63
APEC 02Δ*vir2*	41	29	70.7	0.25	0.81

^A ^As compared to APEC O2.** p < 0.001, no other strains had a significantly different lethality rate than APEC O2

In view of these results, we considered the possibility that chromosomal genes found in APEC O2 may compensate for the function(s) of the lost plasmid genes. For example, many of the genes targeted for deletion in APEC O2 are associated with iron acquisition or complement resistance. Since APEC O2 is known to contain at least two other chromosomal operons (yersinabactin and enterobactin) involved in the acquisition of iron [1], overexpression of chromosomal iron acquisition loci might compensate for the loss of certain plasmid-linked iron acquisition operons. In addition, others have shown that transfer of a ColV plasmid into a K-12 recipient was accompanied by a concomitant increase in the K-12 strain's ability to resist the bactericidal effects of serum complement [33]. However, other studies have demonstrated that when a native host was cured of the ColV plasmid, its ability to survive in serum was not affected [28], suggesting that chromosomal loci may compensate for the lost plasmid genes which conferred complement resistance. Similarly, in our work we did not find any of the mutant derivatives of APEC O2 to be attenuated in their ability to resist complement or to grow under low iron conditions (data not shown).

To further investigate whether chromosomal genes found in APEC O2 were compensating for the deleted plasmid genes, we transferred the mutated pAPEC-O2-ColV derivatives generated above into the AFEC strain NC via conjugation. NC was previously used as a recipient for pAPEC-O2-ColV and was found to have enhanced abilities to kill chick embryos and grow in human urine upon receipt of the plasmids of APEC O2 [3]. The virulence of NC derivatives containing pAPEC-O2-ColV was then compared to that of NC with an intact version of pAPEC-O2-ColV (NC/pAPEC-O2). Again, no differences were found. Knowing that others have found that some of these same genes and regions, such as *tsh *[28] and the *iro *operon [29,34], contribute to the virulence of other APEC, we then reasoned that our virulence assays might be too insensitive to detect subtle changes in virulence caused by the mutations. Indeed, bacterial virulence, as measured with the embryo lethality assay, has been shown to have only a moderate correlation to results of assays done in three-week old chickens [35]. In addition, we and others [36] have not observed a correlation between the infectious dose of a strain and its lethality to chick embryos which would permit comparisons of virulence based on LD_50 _determinations.

Therefore, in an effort to increase the sensitivity of our chick embryo model, we infected 16 day-old chick embryos (the age at which chick embryos are most resistant to *E. coli *infection [36]) via the chorioallantoic route with an equivalent mixture of wildtype and mutant strains (~500 CFUs of each). Three days after infection the surviving embryos (generally > 80% of those infected) were killed by chilling at -20 C for two hours, after which brains, hearts, and livers were collected sterilely. Organ homogenates were cultured quantitatively on agar with or without antibiotics to determine the relative proportions of the strains. The results of these mixed infection experiments partially mimicked our previous embryo lethality data [3]. Strain NC/pAPEC-O2 generally outcompeted strain NC, and no marked differences in organ colonization was seen with any of the mutant strains in relation to the APEC O2 (data not shown). However, we were able to recover *E. coli *from the internal of organs of only a very small (< 15%) proportion of the embryos. This is not entirely surprising as the systemic spread of *E. coli *is not thought to be required for embryo death [36], however it would indicate that mixed infections in 16 day-old chick embryos may have marginal utility in assessing *E. coli *virulence.

**Figure 3:**
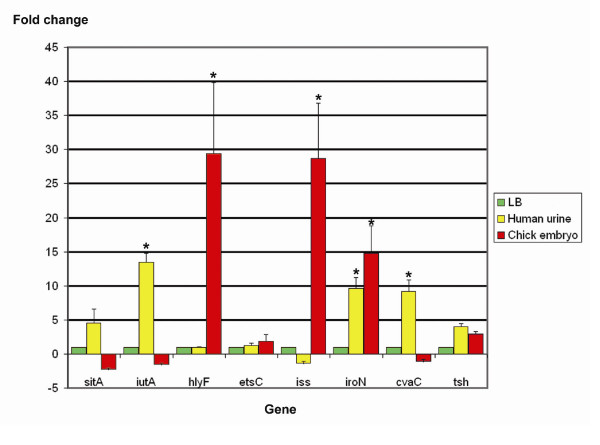
RT-PCR results comparing expression of pAPEC-O2-ColV genes during differential growth conditions. Each bar indicates standard deviation for conditions. Each trial included three replicates, and three biological replicates were performed for each condition. An asterisk above a bar indicates that the difference in expression between growth in LB and in urine or in chick embryos was significant (p-value < 0.05).

Subsequently, we opted to use qRT-PCR in an effort to better understand the nature of pAPEC-O2-ColV's role in APEC O2's growth in human urine and infection of the chick embryo. Here, the genes that had been targeted for mutation plus two additional genes, *hlyF*, encoding an avian hemolysin [10] and *etsC*, a putative ABC transport efflux gene [1], were studied. As compared to exponential growth in LB broth, several genes were up-regulated when grown in human urine or during infection of the chick embryo. In human urine, genes which were up-regulated included *sitA*, *iutA*, *iroN*, *cvaC*, and *tsh*, although *sitA *and *tsh *were not significantly up-regulated (Fig. 3). This was not surprising, as several of these genes have been shown to play a role in iron acquisition [6,8,9], and urine is considered to be an iron-depleted environment [24]. In the chick embryo, different patterns of up-regulation were observed. In general, pAPEC-O2-ColV's iron-related genes were not up-regulated in the embryo, suggesting that the embryo may be a relatively iron-rich environment. Indeed, it has been demonstrated that the percentage of iron in the chick embryo liver is much higher at twelve days of incubation than at hatching [37]. Interestingly, *iss *and *hlyF *were strongly up-regulated (both at approximately 29-fold). *iss *has been shown to play a role in serum  resistance and increased lethality towards day-old chicks [38], while *hlyF *has been shown to exhibit hemolysin activity [10]. Such results suggest that serum resistance and hemolysin production might be important to APEC O2's infection of chick embryos. Also, *iroN *was up-regulated approximately 15-fold. *iroN *has previously been found to play a dual role in *E. coli*'s virulence, serving both as a siderophore receptor and as a virulence factor during urinary tract infection [34]. So too, the results here suggest a possible dual role for *iroN *during avian systemic infection.

The results of this expression analysis are intriguing and suggest that genes localized to ColV plasmids, such as pAPEC-O2-ColV, are involved in the pathogenesis of colibacillosis. The lack of confirmatory results from the mutational analyses illustrates the complexities of such studies, especially where multiple alternative mechanisms may compensate for the deleted genes/operons.

Taken altogether, the results presented here suggest several plausible reasons for an inability to detect significant attenuation in the mutants examined in this study. One possibility is that the embryo lethality assay lacks the sensitivity to detect changes owed to the mutated genes. This is a distinct possibility, as acquisition of pAPEC-O2-ColV by an avirulent recipient confers the ability to kill chick embryos [3] while none of the isogenic mutants created in this study and involving genes and/or regions of pAPEC-O2-ColV were attenuated in the chick embryo model of infection. A second possibility is that none of the deleted genes in this study actually contribute to the abilities of APEC O2 to kill chick embryos or grow in human urine, although this seems unlikely based on the literature, which suggests that several of these genes including *tsh *and *iroN *[28,29,39], do contribute to the virulence of ExPEC. In addition an association of *iss *with the virulence of APEC in a respiratory model of infection has been postulated [40], however a direct role for *iss *in virulence was not demonstrated. In accordance with previous findings, we found that several of these genes were strongly up-regulated during growth of APEC O2 in these models, suggesting that these genes play at least some role in urine growth and infection. To better pinpoint the contributions of these targeted genes and ColV plasmids to APEC virulence, more sensitive virulence assays and use of functional genomics and proteomics approaches examining the total APEC genome will be needed.

## Conclusion

The differences seen in the results of the mutational and transcriptional analyses in this study underscore the need to use multiple approaches in ascertaining genes' contributions to disease.

While the data presented here suggests roles for *iss*, *hlyF*, and *iroN *during *E. coli*-caused septicemia, a more comprehensive analysis of these plasmids is necessary to better understand their nature, and such analysis must also include genes of unknown function found on pAPEC-O2-ColV and other similar plasmids.

## Methods

### Media and bacterial strains

All bacterial strains were stored in Brain Heart Infusion Broth (Difco Laboratories, Detroit, MI) with 20% glycerol at -80 C prior to use [41]. Strains used in this study included NC, APEC O2, and its mutant derivatives. Their relevant characteristics are shown in Table 2.

**Table 2:** Phenotypic Characteristics of Strains/Plasmids Used

**Strain or Plasmid**	**Relevant Characteristics^a^**	**Reference and/or Source**
APEC O2	Virulent to chick embryos; contains pAPEC-O2-ColV and pAPEC-O2-R	Diseased Chicken [1–3]
pAPEC-O2-ColV	ColV plasmid of APEC O2; contains *iss*, *tsh*, and *traT *genes, along with the aerobactin, ColV, *sit *and *iro *operons	[1,3]
pAPEC-O2-R	R plasmid of APEC O2; encodes resistance to multiple antimicrobial agents	[2,3]
NC	Relatively avirulent to chick embryos and lacks *iss*, *tsh*, and the aerobactin, ColV, and *iro *operons	Healthy Chicken [3]
NC/pAPEC-O2	Transconjugant derivative of NC containing pAPEC-O2-ColV and pAPEC-O2-R; has enhanced abilities to kill chick embryos and grow in human urine relative to strain NC	[3]
pKD46	*amp*^R ^; temperature sensitive plasmid which expresses the λ Red recombinase proteins when induced by arabinose	[30]
pSKY5000	*cam*^R ^derivative of pKD46	[42]

^a ^Abbreviations: Amp, ampicillin, Cam, chloramphenicol; ColV, colicin V; r, resistant

### Mutagenesis

Deletions of targeted genes were generated in strain APEC O2 essentially as described by Datsenko and Wanner [30], except that red-mediated recombination proteins were expressed by pSKY5000 rather than pKD46. pSKY5000 is a chloramphenicol resistant (*cam*^R^) derivative of pKD46 [42]. This method relies on the overproduction of λ-derived recombination proteins encoded by the temperature-sensitive plasmid pSKY5000 and PCR amplification of a *kan*^R ^cassette in pKD4 flanked by 5' and 3' sequences of the gene or region targeted for deletion. Cells were electrotransformed, and kanamycin (50 ug/ml) resistant derivatives were identified. After electroporation and *kan *selection, the expected deletions were verified by PCR protocols targeting the deleted gene and the new *kan*^R ^junction fragment [30]. Primers used for mutagenesis are listed in Table 3, and were synthesized at Integrated DNA Technologies (IDT; Coralville, IA).

**Table 3:** Primers used in Mutagenesis*

Name	Primer Seq. (5'-3')	Target(s)*
for-mut-*iutA*	aataatgatgataagcaaaaagtatacgctttgggctctctgtgtaggctggagctgctt	*iutA*
rev-mut-*iutA*	atatcagcgtacctttgttgtaaaggaataccggtcagaacatatgaatatcctccttag	*iutA*, Vir2
for-mut-*tsh*	atactttatgtgcaggcataacggtgctctccctgaattttgtgtaggctggagctgctt	*tsh*, Vir1
rev-mut-*tsh*	ttcttctactgtaccgtaatcagataatcgcagcaaagggcatatgaatatcctccttag	*tsh*
for-mut-*cvaB*	cataagatatatcagccgggaggaaatgagccgatatttttgtgtaggctggagctgctt	*cvaB*
rev-mut-*cvaB*	tcatacgcttgtaattcctctatggtttaaatagaaataacatatgaatatcctccttag	*cvaB*
for-mut-*sitA*	gattgcatcattatttctccctttttccggctttaattcctgtgtaggctggagctgctt	*sitA*
rev-mut-*sitA*	tgctcactataggtactaaattatgcactcaataaaaaaacatatgaatatcctccttag	*sitA*
for-mut-*iss*	tattcatttcccatgattctgagtacctaccaagtctgagtgtgtaggctggagctgctt	*iss*
rev-mut-*iss*	aaaaacaactgtagggagcccagaagtatattaatgaacacatatgaatatcctccttag	*iss*
rev-mut-*iroN*	tattagggaataggtatgagaattaacaaaatcctctggtcatatgaatatcctccttag	*iroN*
for-mut-*iroN*	acgatcagaatgatgcggtaactccggcatagtaagcccgtgtgtaggctggagctgctt	*iroN*
for-mut-Vir2	tgctcactataggtactaaattatgcactcaataaaaaaatgtgtaggctggagctgctt	Vir2
rev-mut-Vir1	tattcatttcccatgattctgagtacctaccaagtctgagcatatgaatatcctccttag	Vir1

* Primers were created using information from GenBank Accession number NC007675.

### Embryo lethality assay

APEC O2 and its mutant derivatives were assessed for lethality in chicken embryos by inoculation of overnight washed bacterial cultures (~500 colony forming units (CFU)) into the allantoic cavity of 12-day old embryonated, specific-pathogen-free eggs [35]. Phosphate buffered saline (PBS) inoculated and uninoculated embryos were used as controls. Embryo deaths were recorded for four days. Differences in embryo lethality between the strains were evaluated for statistical significance using a z-test for the equality of two binomial proportions. P-values of less than 0.05 were considered statistically significant [43].

### Growth in human urine

APEC O2 and its mutant derivatives were compared by their ability to grow in human urine. The assay was performed as described elsewhere [3]. Only urine from healthy, antibiotic-free volunteers, who reported never having experienced a UTI, was used for study. Prior to the study, urine from five volunteers was collected, individually filter sterilized with 0.2 um filters, pooled, and stored at -20 C. On the day before the assay was run, the strains to be tested were grown overnight in 2 ml of Luria Bertani (LB) broth. The next day the cell density was estimated by spectrophotometry, and cultures were diluted in PBS prior to inoculation (100 μl of inoculum into 4.9 ml of urine) to achieve an approximate starting concentration of 10^2 ^to 10^3 ^CFUs per ml, which was confirmed by viable counts. This concentration of bacteria was chosen as a starting point since it represents the lower end of what is considered a significant indicator of UTI in symptomatic young women [44]. Mixtures were incubated at 37 C with shaking, and aliquots of these urine cultures were removed at set time intervals for use in determining viable counts.

### RNA Isolation

Chick embryos were inoculated via the allantoic cavity with APEC O2. Two days later, 12 viable infected embryos were removed from their eggs, and the livers were excised and pooled together in 20 volumes of RNALater (Ambion/Applied Biosystems, Austin, TX). RNA was extracted from these pooled liver samples using Tri Reagent (Ambion), treated with Turbo DNAse (Ambion), followed by phenol/chloroform extraction, and resuspension in distilled water. For *in vitro *isolation, a single colony APEC O2 was inoculated into 3 ml LB Broth or urine and grown at 37° with shaking until the cells were in early- to mid-exponential growth phase (A600 of approximately 0.3). Cells were than pelleted by centrifugation, resuspended immediately in RNALater, and the RNA isolated and purified as described above.

### RT-PCR

Primers for qRT-PCR were designed using software from IDT (Coralville, IA) and also synthesized by IDT. One-step real-time RT-PCR was carried out using an iCycler real-time thermal cycler (Bio-Rad Life Sciences, Hercules, CA) and the iScript One-Step RT-PCR Kit with SYBR Green (Bio-Rad Life Sciences) according to manufacturer's recommendations. Differential gene expression during infection for reference (Glyceraldehyde 3-phosphate dehydrogenase A gene, i.e.,*gapA*) and target genes (Table 4) was calculated using the comparative RT-PCR methods described by Pfaffl [45], where differences in cycle threshold ratios are assessed while accounting for reaction efficiencies. Efficiency and melting curves were generated for each gene assayed. A negative control containing all reagents except reverse transcriptase was included to rule out DNA contamination. Results for each target gene are presented as ratio of expression of that gene in treated cells versus the untreated cells, corrected using the reference gene *gapA *and reaction efficiencies for each gene. Reactions were performed in triplicate, and at least two independent trials were performed for each gene and condition assessed. Standard deviations were calculated for the averages of all of the trials for each gene and condition, and the results plotted. Double-sided p-values were calculated for the relative means using the t-test. P-values of less than 0.05 were considered statistically significant [43].

**Table 4:** Primers used in RT-PCR studies.

Name	Description	Sequence	Predicted amplicon size
gapA F	Glyceraldehyde 3-phosphate dehydrogenase A (reference)	CAT CGT TTC CAA CGC TTC CT	84
gapA R		ACC TTC GAT GAT GCC GAA GTT	
sitA F	Iron/manganese transport gene	TAC GAT CCG GCA AAT GCA CAA ACC	130
sitA R		TGG TGA CCA TCC ATC GCT GAT TCT	
iutA F	Aerobactin receptor gene	TCT GAT AAG AGC GTG GTG GCG AAT	139
iutA R		AGC ACG TTG AAG TTC ACT CCG GTA	
hlyF F	Avian hemolysin gene	AAC TTT GGC GGT TTA GGC ATT CCG	164
hlyF R		TGA CAT ACT GGC AAT GAG CCG TCA	
etsC F	Putative ABC transport gene	ATT ACG AAC AGC GAG TGC TGG AGT	183
etsC R		ATA CGT ACT GCA CCA TGC CGG TAA	
iss F	Increased serum survival gene	GCC GCT CTG GCA ATG CTT ATT ACA	82
iss R		TCC TTT GGT GTT ACT GCT GTC GGT	
iroN F	Salmochelin receptor gene	TTC ACC TGG GAA GAT TAC CAC GCA	109
iroN R		ATA TAT GCG CCT GAA GCG GTT TGC	
cvaC F	ColV structural gene	CGG GCA ATT TGT TGC AGG AGG AAT	111
cvaC R		ACC GGA TGG AGA CAT TGC AGG ATT	
tsh F	Temperature-sensitive hemagglutinin gene	TAC TGA ACC AGC AGG CGG ACA ATA	106
tsh R		TTT ACC TGC CGC TCA TCA GTC AGT	

## Authors' contributions

JAS, TJJ, and LKN conceived and designed the study. JAS and TJJ contributed equally to this work with JAS performing the mutagenesis assays and TJJ performing the transcriptional assays. All authors contributed to the interpretation of the results and writing of the manuscript.
